# A Novel Physical Therapy Approach in Pain Management and Enhancement of Performance in Shin Splints Athletes: A Case Report

**DOI:** 10.7759/cureus.26676

**Published:** 2022-07-09

**Authors:** Nikita S Deshmukh, Pratik Phansopkar, Mayur B Wanjari

**Affiliations:** 1 Musculoskeletal Physiotherapy, Ravi Nair Physiotherapy College, Datta Meghe Institute of Medical Sciences, Wardha, IND; 2 Research and Development, Jawaharlal Nehru Medical College, Datta Meghe Institute of Medical Sciences, Wardha, IND

**Keywords:** conservative management, cupping therapy, pain and function, athletic condition, shin splints

## Abstract

Runners are most commonly attributed to the shin splint, which is showing commonly the symptom of leg pain. It may be misdiagnosed as compartment syndrome as well. This case report depicts the standard condition of medial tibial stress syndrome in a long-distance runner, which is an acute condition with worsening symptoms in many authors' opinion. Patients with accurate symptoms of the conditions may be diagnosed with shin splints for medical usage. Only pain along the posterior medial border of the tibia at the origin of the posterior tibialis muscle should be referred to as shin splints. The chronic form of anterior compartment syndrome may attribute to the runner if they ignore the symptoms of leg pain that occurs in shin splints. Anterior tibial pain during activity is frequent in athletes. It has been linked to various disorders, including periostitis from improper stretching and muscular conditioning, as well as exertional compartment syndromes.

## Introduction

The athlete may get the symptoms of leg pain attributed to shin splints, which may ignore the chronic form of compartment syndrome. According to the American Medical Association's Nomenclature of Athletic Injuries, shin splints are pain and discomfort in the leg caused by recurrent jogging on hard surfaces or violent, extensive use of the foot flexors [1]. Most runners, and even joggers, experience pain around the inner shin boundary at some point. This condition is known as "shin splints" among athletes. The discomfort usually goes away within a few weeks, but it can potentially become chronic, with bouts of pain recurring over months. The discomfort may be so intense that constant training is impossible. Patients have dubbed this illness "medial tibial syndrome" after ruling out the likelihood of a stress fracture [2]. Shin splints are painful inflammations of the tibial periosteum that are most commonly induced by repetitive physical exercise. Repetitive trauma to the tibia can result in partial fractures or microfractures. These tibial stress reactions can make the bone more susceptible to fractures [3]. Due to microtrauma on the tibia, many athletes report pain in the medial side of the shin area. The start of medial tibial stress syndrome (MTSS) is frequently preceded by a period of relative factors contributing to the medial tibial stress syndrome. Unexpected increases in exercise frequency and intensity as well as an underlying weakness, a shin splint is a frequent ailment, ranging from 13.6 to 20% in leisure runners to up to 35% in military people. Shin splints are harmless and self-limiting, and they usually go away after rest. The main consequence of MTSS is the development of stress fracture to the distal third of the tibial bone [4]. During and after prolonged weight-bearing exercises, pain occurs in the front of the leg, which is known as shin splints.

The most common symptom of an overuse injury is discomfort and sensitivity to palpation of the affected area [5]. Shin splints can be caused by various factors, including uneven running surfaces, the degree of conditioning in an athlete, frequent changes in activity level, musculoskeletal abnormalities, running style, and footwear [4]. Running that is forefoot contact running may be the contributory factor of shin splints or contribute significantly to developing the symptoms of shin splints. The forefoot, rather than the heel, makes initial ground contact in forefoot contact running. Several studies have found a link between shin splints and a lack of heel contact [6]. This case report aims to show the efficacy of conventional therapy on a patient with a shin splint. 

## Case presentation

We present a case of a 28-year-old male athlete who reported to the physiotherapy department with a complaint of pain in the area of the shin while playing football with progressive pain. He noticed the pain before the game but ignored it and continued playing. When he was left on the ground, the trainer evaluated him for grade 3 tenderness by grading scale according to Ritchie articular index (AI) and the pertinent finding was mid-shaft tibial tenderness to palpation. The athlete was instructed to rest and apply ice to the affected area. His pain was progressive, so he went to the hospital for urgent care. He went to the orthopedic department for a physical examination and an x-ray. There were no significant findings, so he has been prescribed diclofenac sodium 50 mg, and the orthopedic suggested to do physiotherapy. He did not have any allergies and was not on any other prescriptions. On clinical examination, no abnormal findings were noted. The examination of his right lower extremity revealed severe tenderness grade 3 (Table 1).

**Table 1:** Showing grades of tenderness in the patient before and after treatment.

Tenderness	First week	Second week
Grade	Grade 3	Grade 1

The pain was initiated on movement, and while walking, it was progressive assessed by the visual analog scale (VAS) [2]. The pain was 4/10 on rest, and 6/10 on training before the treatment. After the treatment, the pain was 2/10 on rest and 5/10 on activity after one week of treatment, and the pain entirely subsided after the second week and was 2/10 on activity (Table 2).

**Table 2:** Shows the before and after assessment of pain using VAS. VAS: visual analog scale.

VAS	Pre-treatment	Post-treatment
Score: on movement	6/10	2/10
On rest	4/10	0/10

On the second week of follow-up, the patient's tenderness was reduced from grade 3 to grade 1, as assessed by the tenderness grading scale. There was tightness in the anterior compartment of the leg muscle. Manual muscle testing (MMT) was taken; it was 4/5 for the anterior chamber, 4/5 for the posterior compartment, and 4/5 for the lateral compartment of the leg (Table 3).

**Table 3:** Showing MMT of the right lower extremity before and after treatment. MMT: manual muscle testing.

MMT	Pre-treatment	Post-treatment
Knee flexors	4/5	5/5
Knee extension	4/5	5/5
Ankle dorsiflexion	4/5	5/5
Ankle plantarflexion	4/5	5/5
Invertors and evertors	4/5	4+/5

Active range of motion (AROM) was painful and limited because of pain (Table 4). Assessment of step-up and step-down tests (Table 5) and treadmill tests for the functional evaluation was improved after treatment as compared to before treatment (Table 6).

**Table 4:** Shows the range of motion of the right lower limb before and after treatment. ROM: range of motion, AROM: active range of motion, and PROM: passive range of motion.

ROM	Pre-treatment AROM	Pre-treatment PROM	Post-treatment AROM	Post-treatment PROM
Knee flexion	0-100	0-115	0-130	0-130
Ankle plantarflexion	0-25	0-30	0-40	0-45
Ankle dorsiflexion	0-15	0-20	0-35	0-40
Inversion	0-10	0-15	0-25	0-30
Eversion	0-10	0-15	0-25	0-30

**Table 5:** Shows the assessment of step-up and step-down before and after treatment.

Step-up and step-down test (right leg)	Pre-treatment	First-week assessment	Second-week assessment
Time (termination time)	7 min	12 min	20 min

**Table 6:** Shows the cessation time of the treadmill test before and after assessment.

Treadmill test	Pre-treatment	First week	Second week
Speed	3 mph	9 mph	15 mph
Termination time	5 min	12 min	25 min

Therapeutic intervention

The primary intervention was to switch patients to non-weight-bearing foot contact. The advised was given to the patient to make the leg pain-free first and then start running [6]. Second, to determine whether the patient had learned heel-toe running, his pain was measured again on a treadmill shortly after being given instructions. The patient experienced reduced posteromedial leg pain during the first three days of adjusting his running method. In the first week, he experienced pain in his leg while doing more extended weight-bearing exercises. He was pain-free after only seven days of switching to heel-contact running. He continued to report that he was still pain-free when he ran two weeks later [7]. There was no soreness palpated across the posteromedial tibia or gastrocnemius muscle during the physical examination.

Cupping Therapy

**Figure 1:**
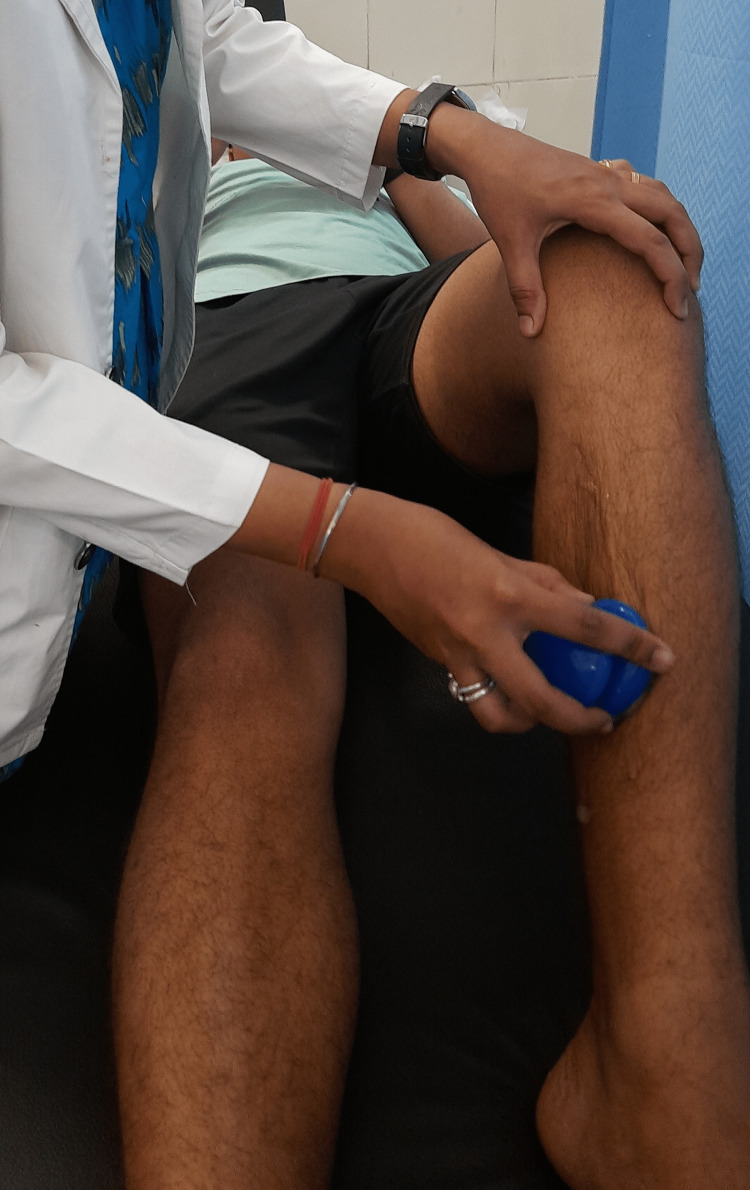
Showed that the cupping therapy was given to the patient with a shin splint.

Cupping therapy can be done using a suction pump or by heating the interior of a glass cup with a flame for a few seconds (Figure 1). Cupping therapy is often performed with a suction pump over the affected area, which increases blood flow and circulation to that muscle. Cupping provides a gentle, sustained stretch for loosening tight muscles and connective tissue adhesion, reducing painful trigger points, improving circulation, and relieving pain. Cupping therapy should not be used if someone has decreased sensation and abnormal bleeding issues. The duration of treatment varies depending on the purpose of treatment and the amount of negative pressure, but it is generally between 1 and 10 minutes [8]. As a result, after a five-minute application of cupping therapy with a silicon cup, the assessment was undertaken in this study. The inside of the silicon cup produces a vacuum for a brief duration. The negative pressure within the silicon cup produces myofascial decompression when placed on the skin [8]. The leg muscle was separated into three parts like anterior, posterior, and lateral and three cupping therapy cups were applied to each area to apply cupping therapy to the affected area of the leg [9].

Stretching

**Figure 2:**
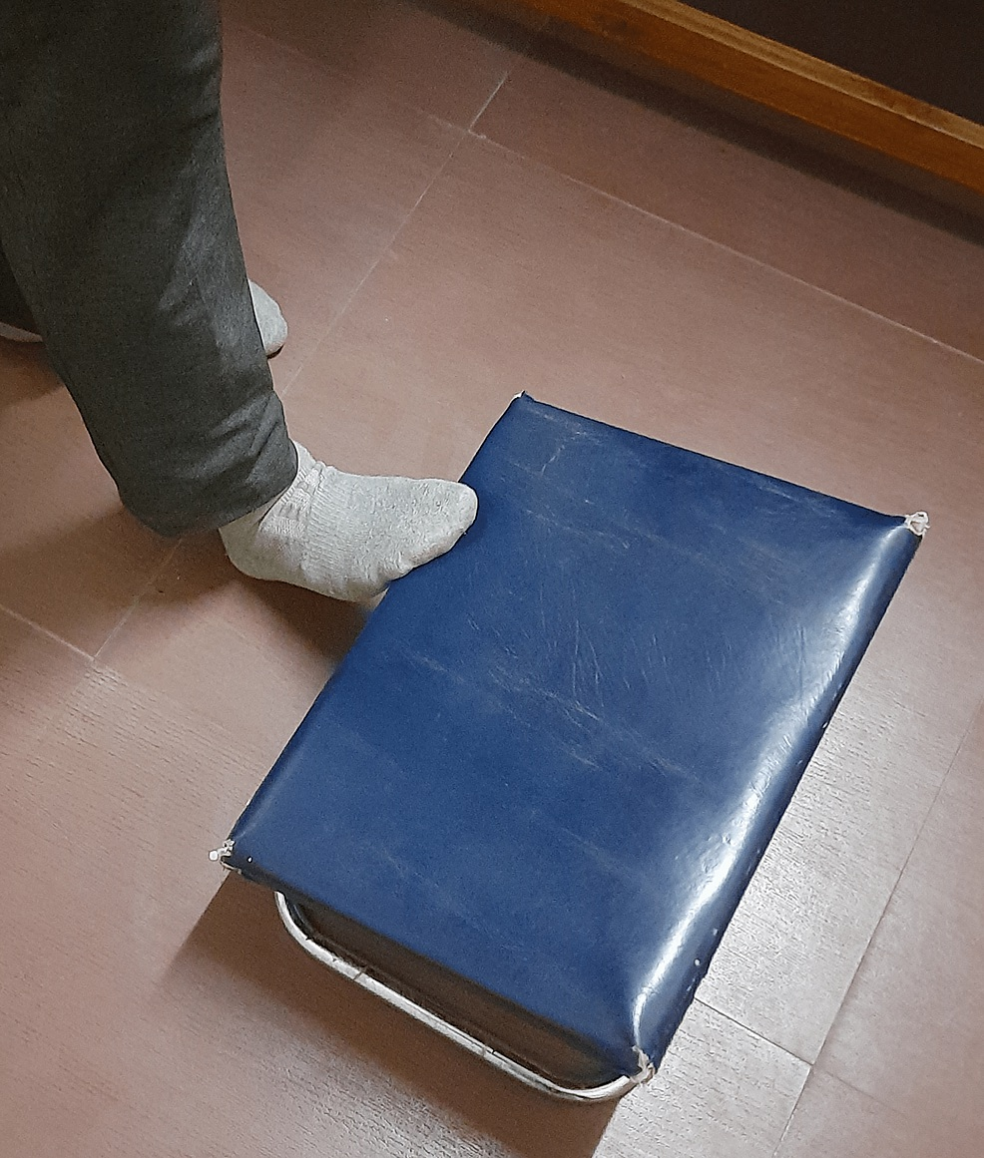
Shows the self-stretching for TA. TA: tendo-achillis.

The subject should be in a standing position for calf stretch, a footstool should be used for stretching purposes, the metatarsal should be on stool, the heel of the patient should be in contact with the ground and ask the patient to lean forward for stretching the calf muscle. The leg was stretched for 10 seconds hold, then slowly returned; this exercise was performed ten times [10] (Figure 2).

Cryotherapy and transcutaneous electrical nerve stimulation (TENS)

**Figure 3:**
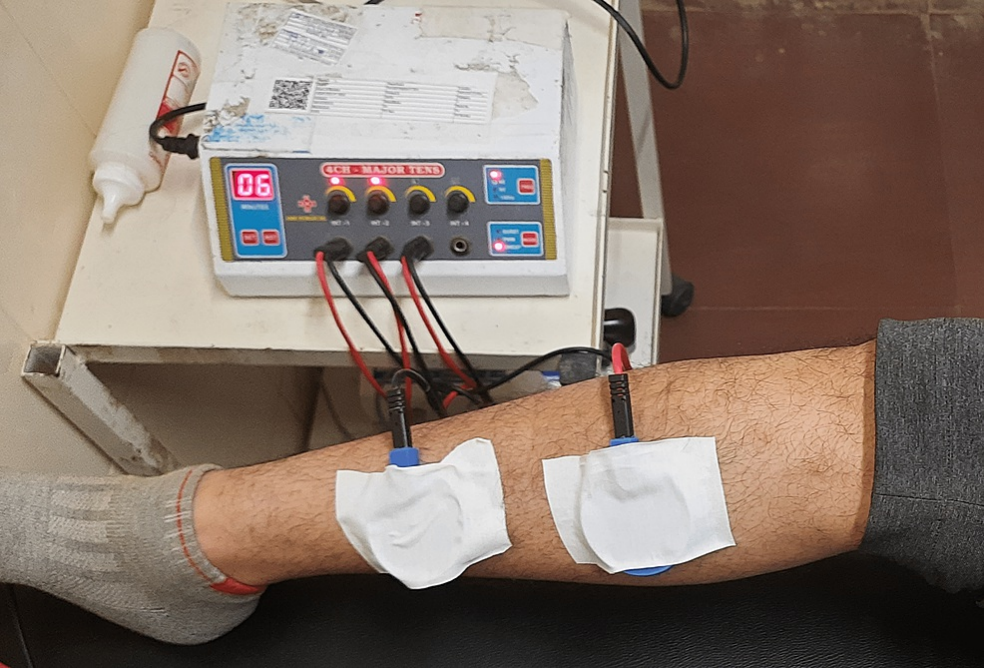
Placement of TENS electrode for shin splint. TENS: transcutaneous electrical nerve stimulation.

Cryotherapy and TENS were used. We are discussing how we apply the TENS machine (Bionics innovations, Mumbai, India) and what are the parameters and placement we use. An ice cube massage was applied to the entire leg for 10 minutes. Then, for an additional 10 minutes, two-channel TENS (pulse rate = 150 Hz, pulse width = 150 s) was applied by using two-electrode of self-adhesive electrodes placed over the anteromedial compartments of the lower leg. The intensity was steadily increased until the participant's comfort level was reached [11] (Figure 3).

Strengthening exercise

Although it appears that acutely injured patients should avoid excessively stretching the triceps surae or engaging in leg muscle strengthening exercises, specific muscle strengthening exercises are frequently prescribed immediately following a diagnosis of MTSS [10]. The anterior tibialis stretch is indicated if anterior leg muscles are involved. It is the same for posterior and lateral leg muscle, which involves placing the foot in plantar flexion and applying mild pressure to the soft tissue in front of the shin while leaning back. The tibialis anterior can be strengthened with painless lower extremity strengthening, and heel lifts will strengthen the gastrocnemius. The strengthening progressed gradually to avoid further aggravation of the tissues. When training resumes, it is critical to follow a proper stretching routine that includes a warm-up and cool-down [12,13]. 

Ice, rest and stretching, strengthening are common treatments for MTSS. Athletes should avoid running on hard surfaces or uneven surfaces, and use proper shoes as well as good sole footwear [14].

## Discussion

Anteromedial shin splints are demonstrated in this case study in which pain occurs in the anteromedial compartment of the leg. Running on uneven surfaces or starting without a warm-up may lead to various injuries [4]. According to Subotnick, forefoot contact running creates much higher ground reaction forces during stance than heel-contact running. Another reason forefoot contact running could cause overuse injuries is that the plantar flexor muscles are constantly contracted throughout the stance [5]. The development of calf discomfort due to shin splints after prolong periods of forefoot contact jogging. Excessive calf muscle contraction is a factor in the development of leg pain. During forefoot contact running, constant dorsiflexor and plantar flexor muscle contractions may have caused posteromedial or anteromedial leg soreness and gastrocnemius muscular tension [6].

MTSS may occur due to prolong running without proper warm-up exercise or not using adequate footwear before athletic activity. Less training or overtraining without formal endurance training and strength training affects the performance or competition performance. Overtraining in any action or exercise leads to a decline in implementation and future injuries [6,10]. MTSS, if not treated early, leads to other complications like affecting the ankle plantarflexion and dorsiflexion. The studies showed that MTSS in the female sex might lead to excessive foot pronation and calf tightness. The case study's outcome is resolving the symptoms without restricting any activity.

We use cupping, TENS, stretching, and strengthening exercise protocols in this case study which may help improve the condition and enhance the performance of athletes. In this case study, many outcome measures are used, and the effect after the treatment or in post-treatment assessment reveals an improvement in symptoms in MTSS conditions. The treatment protocol should not restrict any activity during therapy. However, MTSS is most commonly observed in runners and military populations. It is successfully managed in the acute and early intervention stages and prevents progression.

## Conclusions

Athletes, according to the majority of research, are more likely to get shin splints or medial tibial stress syndrome due to running on hard surfaces. According to the prevalence, this illness affects both men and women equally. Walking, running, dancing, or walking on uneven surfaces are common causes of pain. In this case report, cupping therapy for MTSS has shown significant positive results along with conventional physical therapy interventions such as stretching and strengthening leg muscles. For pain modulation, TENS and cryotherapy proved to be effective. We conclude that a tailored physical therapy program efficiently reduces pain and improves functional capacity and mobility in MTSS.
